# Health and Wellbeing in Higher Education: A Comparison of Music and Sport Students Through the Framework of Self Determination Theory

**DOI:** 10.3389/fpsyg.2020.566307

**Published:** 2020-10-28

**Authors:** Elena Alessandri, Dawn Rose, David Wasley

**Affiliations:** ^1^School of Music, Lucerne University of Applied Sciences and Arts, Lucerne, Switzerland; ^2^School of Sport and Health Sciences, Cardiff Metropolitan University, Cardiff, United Kingdom

**Keywords:** student, wellbeing, higher education, health, Self Determination Theory (SDT)

## Abstract

Students in Higher Education report high levels of mental health issues and psychological distress. Paradoxical findings on performance-orientated students, such as athletes and musicians, suggest that the demands of highly skilled vocations may enhance wellbeing while being detrimental to physical and mental health. To provide timely and appropriate help, institutions need to understand what areas of health and wellbeing are compromised in different student groups. In this study, we compared performance-orientated (music and sport) students to other students and the general population on a selection of wellbeing (WHO5, PWS, and WEMWBS), mental and physical health (K10, SF12, and PHQ9), and trait measures (TIPI, LOT-R, and PCS). Through an online survey (*N* = 273), data were collected from bachelor and master students (*n* = 135 music, *n* = 67 sport, *n* = 71 controls). Students’ scores were compared to the general population, where norm values were available, and analyzed within and between groups. Multiple regression was performed to investigate trait measures as predictors of wellbeing. All groups scored significantly below population norms for wellbeing and mental health. One third were classed as having moderate to severe depression. Musicians scored higher openness to experience than athletes. While sport students showed a highly homogenous within-group profile, music students’ scores differed significantly across study courses (e.g., performance and composition). Predictors for wellbeing were: optimism and emotional stability (all students); additionally conscientiousness (sport and music); and perceived competence (music only). As expected, students reported more health and wellbeing issues than general population. Distinct profiles of wellbeing were apparent for performance-orientated students. Results are in line with Self Determination Theory and suggest the need for institutions to embed health and wellbeing into a ‘living curriculum’ to accommodate the needs of different student groups. The WHO5 emerged as a parsimonious yet sensitive measure for mental health and wellbeing in student populations.

## Introduction

Tertiary education encompasses two major transitional stages in life; firstly, moving from school toward self-directed learning and from ‘home’ toward independent living, followed by preparation for professional life, often through developing interpersonal competencies and adult life skills (Malebo et al., 2007; Hewitt, 2019). These changes are thought to impact directly and indirectly on the economic, social, physical and psychological aspects of health and wellbeing of students (Nissen et al., 2019). In general, studies of students in higher education (henceforth: HE) have reported higher levels of mental health problems relative to the general population (Neves and Hillman, 2019). Disabling symptoms include high levels of anxiety, depression and psychological distress, in turn lowering grade average and increasing student dropout (for review see Storrie et al., 2010). However, according to Self-Determination Theory, which is based on a eudaimonic perspective of wellbeing, challenging periods are not necessarily detrimental to wellbeing, and can instead enable personal growth and feelings of achievement (i.e., eustress rather than distress) depending to some extent on mindset (Dweck, 2000; Deci and Ryan, 2012).

These two perspectives on student welfare, namely *mental health* and *wellbeing*, whilst not unrelated, can be conflated when studies measure one aspect but infer outcomes related to the other. A recent report conducted for the Higher Education Policy Institute (HEPI), United Kingdom (Hewitt, 2019) suggested this is problematic with regard to the provision of the correct resources (student support networks and services, and interventions for example). The HEPI report suggests using a two-continua model of wellbeing which includes both mental health and wellbeing to avoid conflation of the two constructs when considering student welfare.

Historically the term *mental health* has been associated with a psychiatric/medical model (with a negative value-laden inference) whereby treatment is administered to improve mental illnesses with clinically significant symptoms, such as depression (Wren-Lewis and Alexandrova, in press). Current data relating to student mental health, and associated stigma, suggests the use of this term remains relevant as it is indubitably tied to measures focused on physical and mental health problems linked to psychological distress (Storrie et al., 2010; Spahn et al., 2017; Hughes et al., 2018; Nissen et al., 2019).

In contrast, the term wellbeing, though achieving no consensus of definition according to the Centers for Disease Control and Prevention (2018), has been adopted as a more positive and holistic approach to subjective life experience and not merely the absence of disease or infirmity (WHO, 1948). The term wellbeing can include both hedonistic (enjoyment of positive affective states, happiness, and pursuit of pleasure) and eudaimonic (experiencing meaning and purpose in life) perspectives of wellbeing (Dodge et al., 2012). The eudaimonic perspective accords with Self-Determination Theory, a framework often used in education in relation to self-realization (Ryan and Deci, 2001). Dodge et al. (2012) suggest that as a definitive definition of wellbeing remains elusive, wellbeing can be operationalized as ‘the balance point between one’s resource pool and the challenges faced’ (p. 230). This approach is salutogenic rather than medical, focusing on how individuals comprehend, manage and make meaning of their lives even when faced with health challenges (Antonovsky, 1979; Sidell, 2007).

Accordingly, in the context of HE for this paper, the eudaimonic perspective of wellbeing was embraced according to the definition provided Dodge et al. (2012), and operationalized to include measures which capture global and factorial aspects of eudaimonic wellbeing in comparison to measures intended to capture mental health difficulties in accordance with the medical model. Mental ‘health’ tends to be conceptualized negatively as mood or anxiety disorders that could be diagnosed according to the Diagnostic and Statistical Manual of Mental Disorders Fifth Edition (DSM-5; American Psychiatric Association, 2013) in accordance with Castillo and Schwartz (2013), and operationalized using measures which specifically focus on capturing data related to mental health, depression, anxiety and psychological distress. Consequently, the term *health and wellbeing* is used throughout to reflect this suggested approach.

In order to enable the planning of appropriate support systems, institutions need to know which areas of student health and wellbeing are generally compromised, and which student groups are in need of specialized, targeted, and appropriate care (Hunt and Eisenberg, 2010). In the present study, we focus on the health and wellbeing of students in ‘performance-orientated’ vocation-based subjects, such as sport and music students, in which there is an embodied element of expertise in comparison to general university courses (i.e., other students). This is because research has suggested that the nature of demands involved in ‘performance-orientated’ and embodied areas of study, like music and sport, may paradoxically enhance wellbeing (e.g., Malebo et al., 2007; Ascenso et al., 2018a, b; Philippe et al., 2019), and/or be detrimental to physical and mental health (e.g., Bartholomew et al., 2011; Hildebrandt et al., 2012; Spahn et al., 2017; Kouali et al., 2020).

Subjects such as music and sports, which often involve immediate feedback in terms of formal and informal evaluation by experts and lay-audiences alike, require a long-term commitment to deliberate practice and improvement prior to HE. However, different course types may have different demands in terms of performance orientation. Studying music theory and/or composition may be more solitary endeavors for example, compared to music/sport performance and/or pedagogy which may entail a continuum of social factors from individual practice/training and/or solo performance, through to ensemble/team playing and onto direct individual and/or group competition. Such dedication to excellence, where finding the balance between distress and eustress is critical, has been the subject of much investigation in relation to expertise (e.g., Ericsson et al., 2018). On the one hand, the thousands of hours of preparation, training and practice, the pressure of strenuous travel and concert/competition schedules, and the stress of optimal performance (i.e., performing regularly at high-levels under performance-evaluative conditions) in combination with uncertainty about the future, all put a strain on the mind and body of musicians and athletes (e.g., Smith et al., 2009; Williamon et al., 2009; Spahn et al., 2017; Kouali et al., 2020). Such ‘devotion’ can lead to vocation-related problems (such as musculoskeletal injuries, see e.g., Leaver et al., 2011; Kenny and Ackermann, 2015; Goossens et al., 2019) and has been associated with higher levels of anxiety, depression and psychological distress (Steptoe, 1989; Kenny and Osborne, 2006; Vaag et al., 2014; Gouttebarge et al., 2015). Furthermore, stigma (i.e., the cultural perception of ‘weakness’ in music and sport contexts) may present particular barriers that prevent expert performance students from seeking help (Nicholls et al., 2008; Spahn et al., 2017).

On the other hand, musicians and sports people may develop healthy behaviors and adaptive coping strategies during their training (Lundqvist, 2011; Korte et al., 2020). Such psychological capital could be augmented, for example, by the development of *autotelic* values, as observed in musicians (i.e., goal-directed enjoyment of learning, see Elliott, 1993), leading to a high level of *functional autonomy* (Allport, 1961) that may prepare them for independent learning and living. It has been suggested that the increased contact time between music and sport students, teachers, parents, and peers was also socially beneficial, leading to increased self-esteem, which in turn led to higher motivation and feelings of self-efficacy (Broh, 2002; Sheridan et al., 2014).

Certain personality traits may also underpin the efficacy of coping mechanisms (Eysenck et al., 1982). Having high levels of dispositional optimism, for example, has been shown to provide protective qualities against stress for students in general (Vickers and Vogeltanz, 2000; Schulz et al., 2009; Mazé and Verlhiac, 2013). Being optimistic is a characteristic associated with mental toughness in sport students (Nicholls et al., 2008) and has been framed as providing a buffer against stressful challenges for musicians (Roy et al., 2016; Orejudo et al., 2017).

Researchers have also considered the role of the big five personality traits in relation to the ability to cope with life stressors for students in terms of either group comparisons, or in relation to normative data depending on the study. A review by Tosevski et al. (2010) suggests that students with high levels of conscientiousness for example, have a higher tendency toward social phobia, which in turn has been directly associated with mental health problems. In contrast, students with high levels of extraversion are more likely to have mature coping styles that are associated with good mental health. Studies of musicians have consistently found higher levels of the personality trait of ‘openness to experience’ (Vuoskoski and Eerola, 2011; Müllensiefen et al., 2014; Greenberg et al., 2015; Rose et al., 2019). However, this trait has been associated more with creativity than adaptive coping strategies for health and wellbeing *per se*.

Although instinctively it is assumed that musicians have high levels of extraversion, in fact it is sports people who have been found to exhibit higher extraversion, and less neurosis (i.e., higher emotional stability) than comparative groups (Rhodes and Smith, 2006; Joshanloo et al., 2012). A recent study provided evidence that neuroticism negatively predicted coping efficacy in athletes (Kaiseler et al., 2019). When reviewing early research, Eysenck et al. (1982) suggested that differences should be apparent according to the nature of individual and team sports, an idea that lends itself to the question of whether a similar pattern between solo and ensemble musicians would be evident.

Studies on student wellbeing commonly utilized a variety of wellbeing or health measures both singularly and in combination. Wellbeing measures have included various World Health Organization (WHO) measures (Helou et al., 2019; Philippe et al., 2019), the Warwick and Edinburgh Mental Wellbeing Scale (WEMWBS; Harding et al., 2019; Kidger et al., 2019), the Positive emotion, Engagement, Relationship, Meaning and Accomplishment (PERMA) profiler (Ascenso et al., 2017; Smith et al., 2020), the Satisfaction with Life scale (Samaranayake and Fernando, 2011; Moate et al., 2019) and various versions of Ryff’s Psychological Wellbeing Scale (PWB; Chraif and Dumitru, 2015; De Clercq et al., 2019). Unidimensional mental health measures have included the Beck depression and anxiety inventories (Yazici et al., 2016; Yüksel and Bahadir-Yilmaz, 2019), the Depression Anxiety Stress scale (Koops and Kuebel, 2019), and the Kessler scale of psychological distress (K10; Brewer et al., 2019). Multidimensional measures used were, for example, the General Health Questionnaire (Bore et al., 2016), the Personal Health Questionnaire (PHQ 9; Lipson et al., 2016), and the SF12 (Wilks et al., 2020). Other studies have explored traits in students, like personality (Joshanloo et al., 2012), measures of mindset (Ortiz Alvarado et al., 2019) and perceived competence (Levesque et al., 2004), dispositional optimism (Morton et al., 2014) and mental toughness (Nicholls et al., 2008). With few exceptions, like Spahn et al. (2004), no study so far has investigated wellbeing comparing different groups of performance-orientated students. The variety of measures used and the paucity of comparative studies makes comparing findings and the development of an overall understanding of student wellbeing difficult (Scott and Takarangi, 2019). Yet, with only one third of students seeking help in an appropriate and timely manner (Macaskill, 2013), it is essential for institutions to develop tools and understanding that enable targeted prevention and wellbeing promotion strategies.

As a step in this direction, the present study compared ‘performance-orientated’ (i.e., music and sport) and other students in a specific subject on a selection of wellbeing, physical and mental health, and trait measures. Our aim was twofold: first, to consider which measures best capture *health and wellbeing* in students with a focus on expert performance in a parsimonious way, while still reflecting the diverse nature of different student groups (i.e., context-specific wellbeing, Daniels, 2000). Second, to explore similarities and differences between student groups, thus clarifying areas of wellbeing or trait patterns that are unique to ‘performance-orientated’ students.

## Methods and Measures

### Participants

Participants for this study included undergraduate (bachelor) and postgraduate (master) students aged between 18 and 30 years old. The total number of participants was 273, including students who described themselves as Bachelor *n* = 202 (74%), Master *n* = 65 (24%), or who were studying an Alternative Professional Qualification (such as at a music conservatoire) *n* = 6 (2%). The Mean Age of the students was 22.22 years, *Median* = 21, *SD* = 3.06. The participants identified as Female; *n* = 192 (70%), Male; *n* = 76 (28%), and Other Gender ID; *n* = 5 (2%). Countries of residence of the participants are depicted in Table 1.

**TABLE 1:** Participants’ country of residence.

Country	*n*	%
United Kingdom	108	40
Switzerland	101	37
Germany	45	17
Pakistan	5	2
Canada	4	2
Italy	3	1
Netherlands	1	<1
Austria	1	<1
Belgium	1	<1
France	1	<1
New Zealand	1	<1
Nigeria	1	<1
United States	1	<1

Groups included Music students (*n* = 135, 49%), Sport students (*n* = 67, 25%), and other students as controls (*n* = 71, 26%). Music and Sport groups were classified according to the type of course and type of performance through forced choice questions (see Table 2).

**TABLE 2:** Participant demographics and group characteristics.

	Music (*n* = 135)	Sport (*n* = 67)	Other (*n* = 71)
**Age**			
Mean age	22.23	20.90	23.44
Mean age *SD*	3.17	2.04	3.18
Median age	21	21	23

**Gender identification**	***n* (%)**	***n* (%)**	***n* (%)**

Female	101 (75)	39 (58)	52 (73)
Male	30 (22)	28 (42)	18 (25)
Other	4 (3)	–	1 (1)
**Level of higher education**			
Undergraduate/bachelor	88 (65)	56 (84)	58 (82)
Postgraduate/master	42 (31)	11 (16)	12 (17)
Alternative professional qualification	5 (4)	–	1 (1)
Nature of music course			
Music performance	74 (55)	–	–
Composition/song writing	18 (13)	–	–
Music theory/research	16 (12)	–	–
Music pedagogy	27 (20)	–	–
**Nature of music performance**			
Solo	27 (20)	–	–
Ensemble/group/orchestra players	39 (29)	–	–
Both solo and ensemble	65 (48)	–	–
Currently not playing music due to injury	2 (1.5)	–	–
Missing data	2 (1.5)	–	–
**Nature of sport course**			
Training/competing/coaching	–	22 (33)	–
Sport science and research	–	38 (57)	–
Sport pedagogy	–	7 (10)	–
Nature of sport performance			
Individual	–	25 (37)	–
Team	–	30 (45)	–
Both individual and team	–	7 (10)	–
Currently not playing sport due to injury	–	4 (6)	–
Missing data	–	1 (2)	

In addition to these demographic items, the survey also asked (forced choice) students whether they (a) currently suffered with a physical injury related to their chosen course (Y/N), (b) were currently taking medication (Y/N, and if so, a free choice description) and (c) the nature of the parental support they experience (positive/supportive, neutral, or negative/conflict). Overall, *n* = 41 (15%) of students reported having a physical injury related to their course, and *n* = 65 (24%) reported currently taking medication that had been prescribed. With regard to the nature of parental support, *n* = 213 (78%) students reported experiencing positive support, *n* = 53 (20%) reported a neutral experience and *n* = 7 (3%) reported negative experience of conflict with parents regarding their chosen course.

### Statement of Ethics

Ethical approval for the online study was granted by the Ethics Committees of Cardiff Metropolitan University/Prifysgol Metropolitan Caerdydd in the United Kingdom. At the beginning of the study participants were asked to confirm they could read and understand English, had read the information sheet provided, and were currently engaged in higher education before giving consent for their anonymized data to be used in the study for academic purposes. Prior to participating, students were informed that for each completed survey, £2 would be donated to the Student Minds charity (https://www.studentminds.org.uk). The website for this charity was provided for participants as an online resource for further assistance should they require such services. At the end of the survey, students were also notified that, as further personal compensation for their time, and to help with their future studies, they could choose to be entered into a prize draw for a Lenovo Yoga 2-in1 computer (one winner). Students were asked to leave an email contact for this purpose, or if they wished to be notified about the results of this study, or would like to participate in future studies. When asked if they would like to take part in future research, *n* = 133 (49%) of these participants said they would. When asked if they would like to be contacted about the results of this study, *n* = 148 (54%) of these participants said they would. When asked if they would like to be entered into the prize draw, *n* = 165 (60%) of these participants said they would.

### Measures

The measures were administered in the following order: Perceived Competence Scale (PCS), Psychological Wellbeing Scale (PWB), Revised Life Orientation Test (LOT-R), Warwick–Edinburgh Mental Wellbeing Scale (WEMWBS), Personal Health Questionnaire (PHQ9), World Health Organization Wellbeing Index (WHO-5), Physical and Mental Health Summary Scales – Short Form (SF12), Ten Items Personality Inventory (TIPI), and Kessler Measure of Psychological Distress (K10). Three wellbeing measures (WHO-5, WEMWBS, and PWB) and three health measures (K10, SF12, and PHQ9) were selected to cover student health and wellbeing components. In addition, three individual trait measures (TIPI, LOT-R, and PCS) were used to explore possible mediating factors.

#### Wellbeing Measures

(1) World Health Organization Wellbeing Index (WHO - 5, Bech et al., 2003). The WHO-5 is a brief generic global rating scale of subjective wellbeing (Topp et al., 2015). The five statements are positively worded and scored using a 6-point Likert scale (*All of the time* to *At no time*) for a time scale related to the past 2 weeks. Raw scores (ranging from 0 to 25) are transformed to percentage scores (as used in this study) by multiplying by four, whereby 0 represents the worst imaginable wellbeing compared to 100 which represent the best imaginable wellbeing. The total mean score in this study was based on data from participants in thirteen countries. To provide the most closely aligned comparator score from the population norms published in Supplementary Table 2 in Topp et al. (2015), the mean of the mean scores of seven of the thirteen countries mean was used. The Cronbach’s alpha coefficient reported for the WHO-5 is >0.80 (e.g., Garland et al., 2018; Ismail et al., 2018). Whilst the WHOQOL-BREF (1998) has previously been used in studies of music students (Philippe et al., 2019) and sport (Verkooijen et al., 2012; Yazicioglu et al., 2012), to the best of our knowledge, this very short version has not.

(2) Warwick–Edinburgh Mental Wellbeing Scale (WEMWBS; Tennant et al., 2007). The WEMWBS was developed to measure individuals’ state of mental wellbeing (thoughts and feelings) during the last 2 weeks. The measure is a 14-item scale, scored using a 5-point Likert agreement scale (*None of the time* to *All of the time*). Statements are worded positively and designed to include both hedonic and eudaimonic aspects of psychological functioning. Scores range from 14 to 70, population scores approximate to a normal distribution without ceiling or floor effects. Based on the 2006 dataset (Stewart-Brown et al., 2008), the normative score used in this study was 52, the mean of the mean scores for 16 to 24-year olds (*n* = 176, *Mean* = 53) and 25 to 34-year olds (*n* = 245, *Mean* = 51). The Cronbach’s alpha coefficient for the WEMWBS is 0.89.

(3) Psychological Wellbeing Scale (commonly referred to as PWB; Ryff, 1989; Ryff et al., 2017). Ryff’s (1989) concept of wellbeing is more philosophical than medical in nature, encompassing multi-dimensional constructs related to trait eudaimonic wellbeing, including wellness of body and mind enabling engagement in living. The 42-item (7 items per scale) version of the PWB, scored with a 6-point Likert agreement (*Strongly disagree* to *Strongly agree*) does not offer a global score. Instead, this was used to capture the six dimensions of wellbeing separately; Autonomy, Environmental Mastery, Personal Growth, Positive Relationships with Others, Purpose in Life, and Self-acceptance. Internal consistency for the six scales range from 0.82 to 0.90 (Schmutte and Ryff, 1997). The PWB statements are worded to establish the participants feelings about their current state of wellbeing.

#### Physical and Mental Health Measures

(1) Personal Health Questionnaire (PHQ9; Kroenke et al., 2001). The PHQ9 is a 9-item questionnaire used to screen for the presence and severity of depression according to the diagnostic criteria identified in the Diagnostic and Statistical Manual of Mental Disorders (DSM-5; American Psychiatric Association, 2013). The time-scale of the items is related to the individual’s experience during the past 2-weeks. Items are scored with a 4-point Likert agreement scale (*Not at all* to *Nearly every day*). Scores range from 0 to 27, and responses suggest varying levels of depression classed as so: 0–4 = none/minimal, 5–9 = mild, 10–14 = moderate, 15–19 = moderate/severe, 20–27 = severe. These classifications were used to depict levels of depression for analysis in this study. The Cronbach’s alpha coefficient for the PHQ9 is 0.89.

(2) Kessler (K10) Measure of Psychological Distress (Kessler et al., 2002). The K10 is a global measure of psychological distress representing feelings of anxiety and depression over the previous 4 weeks. The ten statements are scored on a 5-point Likert scale cores (ranging 10–50). Scores are then classified as <20 = likely to be well, 20–24 = likely to have a mild mental disorder, 25–29 = likely to have a mild moderate mental disorder, and 30 or more = likely to have a severe mental disorder. These classifications were used to depict levels of psychological distress for analysis in this study. The Cronbach’s alpha coefficient for the K10 is 0.93.

(3) Physical and Mental Health Summary Scales - Short Form (SF12; Ware et al., 1994). The SF12 represents eight common concepts of health; physical functioning, role limitations due to physical health problems, bodily pain, general health, vitality (energy/fatigue), social functioning, role limitations due to emotional problems, and mental health (psychological distress and psychological wellbeing). Scoring algorithms are complex but items scores can be entered into a freely available website^1^ which computes scores for physical component summary and mental component summary health separately. The time period of the questions can be between 1 and 4 weeks. Based on a sample of the general United States population (*N* = 2,333), algorithms were used to generate normative data from a longer form (SF36) for the SF12, providing an average score of 50 for each factor. These norms were tested against smaller samples of the general United States (*n* = 232, McHorney et al., 1994) and United Kingdom (*n* = 187, Brazier et al., 1993) populations. The Cronbach’s alpha coefficient reported for the Physical components were 0.89 and 0.86 in the United States and United Kingdom, respectively, and for the Mental component, 0.76 and 0.77, respectively.

#### Individual Traits

(1) Perceived Competence Scale (PCS, Williams and Deci, 1996; Williams et al., 1998). Competence, a concept akin to self-efficacy (Bandura, 1982), is assumed to be one of three fundamental psychological needs (the others are autonomy and relatedness) within the framework of Self-Determination Theory (SDT). Competence can be thought of as a “subjective sense of capability” (Matin et al., 2019, p. 2). Having higher feelings of competence is thought to be related to having a sense of life-satisfaction, and therefore eudaimonic wellbeing (Deci and Ryan, 2012). The PCS is a 4-item questionnaire scored with a 7-point Likert agreement scale (*Not at all true* to *Very true*). Items on the PCS are typically edited to be specific to the relevant behavior or domain being studied (see Supplementary Material A for full survey wording). The score of the four items are averaged with the mean used as the final score for comparison between groups, or for change over time studies, rather than against a population norm due to the context-related wording of the items. Nevertheless, Cronbach’s alpha have been reported as above 0.80 in a number of studies (e.g., Williams and Deci, 1996; Matin et al., 2019).

(2) Life Orientation Test (Revised; LOT-R, Scheier et al., 1985, 1994). The revised life orientation test (LOT-R) is a trait measure of how people approach the world, characterized depending on whether they believe good (optimism, positive bias) or bad (pessimism, negative bias) things will happen to them (Scheier and Carver, 2003). Research suggests these expectancies are linked to wellbeing: optimism is helpful for overcoming physical and psychological difficulties (adaptive skills, Scheier and Carver, 1993), whereas pessimism has been associated with higher levels of depression, poor physical health, lowered immune function (Dember et al., 1989) and less effective coping strategies (Carver et al., 1989). The six statements (three items for each construct) are worded for the present and scored using a 5-point Likert scale (*I agree a lot* to *I disagree a lot*). Studies have reported Cronbach alpha of 0.70–0.71 for optimism and 0.68–0.74 for pessimism (Herzberg et al., 2006; Glaesmer et al., 2012).

(3) Ten Item Personality Inventory (TIPI; Gosling et al., 2003). The TIPI is a brief inventory of personality traits based on the Big Five framework (McCrae and Costa, 1987; John and Srivastava, 1999). The 10 statements are scored on a 7-point Likert scale (*Disagree strongly* to *Agree strongly*) whereby score range 2–14 for the two items per construct. No time frame is provided in relation to the statements. The Cronbach alphas statistics reported are quite low; Extraversion = 0.68, Agreeableness = 0.40, Conscientiousness = 0.50, Openness to Experience = 0.45 and Emotional Stability = 0.73, which the authors explain as an example of how validity can exceed reliability. Instead, the authors suggest using the test–retest reliability coefficients as reliability estimates (Overall = 0.72, Extraversion = 0.77, Agreeableness = 0.71, Conscientiousness = 0.76, Openness to Experience = 0.62, and Emotional Stability = 0.70).

### Procedure

Data were collected from 13th September 2019 until the 10th February 2020 exclusively via online survey (Supplementary Material A) using the survey platform, Qualtrics^2^ (UT, United States). To ensure anonymity, the settings on the survey software were programmed to the highest level of anonymity; that is no personal details were collected, and participant’s IP addresses were not recorded. Recruitment took place via social media as well as e-networks (such as email invitation) developed by the researchers, for example through their teaching experience and/or training as musicians and in sports. The survey began with a description of why the study is being undertaken and by whom (including contact details). Participants were then assured that the study had been granted ethical approval by the appropriate authorities and were provided with a link to a full Participant Information Sheet. Participants were then asked to provide demographic information, and then to answer the selected wellbeing, health, and traits measures. At the end of the survey, participants were offered the opportunity to be informed of the results, take part in future research, and/or enter a prize draw, and were reminded that a small financial donation would be made on their behalf to a charity focusing on student wellbeing. The participant was thanked for their time and reminded of whom to contact before the survey ended.

## Results

### Data Preparation and Analysis

In total, 469 students participated in the online survey, of which 72% completed up to 60% of the survey. Students who completed less than 60% of the survey, who were over 30 years old, and who were studying at doctoral level were not included in the analyses (due to evidence that different types of stressors are prevalent, Ortiz Alvarado et al., 2019), resulting in the final total sample of 273 students. Those students completed the survey in 25 min on average.

Analyses were conducted comparing student groups to population norms, between all three groups, between music and sport (‘performance-orientated’) groups, and within music and sport groups. In addition, correlation analyses were used to explore relationships between the three wellbeing measures as well as between wellbeing and other measures (health, traits). When assumptions for parametric analyses are met, ANOVA and t tests were used as appropriate. Due to the unequal sample sizes, non-parametric analyses were conducted where appropriate, i.e., Kruskal–Wallis for more than two groups. Follow up tests were pairwise comparisons and effect sizes were calculated as so: for *t*-tests, Cohen’s *d* (small = 0.2, medium = 0.5, large = 0.8), for ANOVA partial eta squared ($\eta_{\text{p}}^{2}$) is provided as an estimate of variance explained within the sample. For Kruskal–Wallis tests, significant *post hoc* pairwise comparisons, *z* scores were converted to *r* (small = 0.1, medium = 0.3, large = 0.5; Field, 2018, pp. 117, 318). To avoid confusion with correlational analyses, effect size is denoted as *ES* where relevant in text. Significance level alpha *p* is set at *p* < 0.003 adjusted for multiple comparisons. Multiple regression analyses were conducted to ascertain the predictive value of perceived competence, dispositional optimism and personality traits on wellbeing by group. Pearson’s bivariate correlational analyses (two-tailed) were conducted between measures.

Table 2 shows the sample characteristics. Means and standard deviations for all measures are supplied in Supplementary Material B. To further characterize the sample in relation to their psychological health, two of the scales (K10 and PHQ9) include threshold criteria which allows for preliminary classification of individuals. According to the K10 measure of psychological distress (*n* = 16 missing scores), 126 (46%) participants scored within the lowest range (likely to be well), *n* = 50 (18%) were likely to have a mild disorder, *n* = 40 (15%) were likely to have a moderate disorder, and *n* = 41 (15%) were likely to have a severe disorder. Similarly, for the PHQ9 (*n* = 5 missing scores), although *n* = 75 (28%) students were classed has reporting no symptoms of depression, *n* = 100 (37%) were classed as having mild depression, *n* = 53 (19%) as having moderate depression, *n* = 22 (8%) as having moderate to severe depression, and *n* = 18 (7%) as having severe depression.

### Inferential Statistical Results – Student Groups Compared to Population Norms

The results presented in Table 3 report group means against population norms for the fours scales (WHO-5, WEMWBS; SF12 physical and mental health) where such data is available (mostly age). As shown, all three student groups scored significantly below normative population data, with large effect sizes, for three out of the four measures of health and wellbeing.

**TABLE 3:** Student group comparisons to population norms for standardized scales of health and wellbeing.

Measure	Pop. *Mean*	Group *n*	Group *Mean*	*SD*	*SE*	Statistic	*p*-value	Effect size (*d*)	Mean Diff.	Confidence intervals
**World Health Organization (WHO-5)**	63.6									
Music		130	52.74	20.27	1.78	*t*_(129)_ = −6.11	*p* < 0.001	0.55	−10.86	−14.38 to −7.34
Sport		65	55.82	22.51	2.79	*t*_(64)_ = −2.79	*p* < 0.01	0.49	−7.79	−13.36 to −2.21
Other		70	48.63	20.13	2.41	*t*_(69)_ = −6.22	*p* < 0.001	0.56	−14.97	−19.77 to −10.17
**SF12 Physical health**	50									
Music		123	49.98	9.37	0.85	ns	*p* > 0.9	–	–	–
Sport		62	49.65	9.28	1.18	ns	*p* > 0.7	–	–	–
Other		70	51.53	8.93	1.07	ns	*p* > 0.2	–	–	–
**SF12 Mental health**	50									
Music		123	42.18	13.36	1.21	*t*_(122)_ = −6.49	*p* < 0.001	3.09	−7.82	−10.22 to −5.44
Sport		62	41.44	13.33	1.68	*t*_(61)_ = −5.10	*p* < 0.001	3.09	−8.57	−11.92 to −5.21
Other		70	40	12.99	1.55	*t*_(69)_ = −6.44	*p* < 0.001	3.17	−10	−13.1 to −6.90
**Warwick–Edinburgh Wellbeing Scale**	52									
Music		135	47.03	9.61	0.83	*t*_(134)_ = −6.01	*p* < 0.001	4.87	−4.97	−6.61 to −3.33
Sport		67	46.04	10.42	1.27	*t*_(66)_ = −4.68	*p* < 0.001	4.49	−5.96	−8.50 to −3.41
Other		71	47.24	9.96	1.18	*t*_(70_) = −4.03	*p* < 0.001	4.70	−4.76	−7.12 to −2.46

### Inferential Statistical Results – Between Groups

Statistical significance level (adjusted for multiple comparisons according to Bonferroni method) was *p* < 0.003. Non-significant results (*ns*) are provided in brackets for full reporting.

The results of all between-groups analyses are presented in Table 4. Significant differences were found for demographics and trait measures. A significant main effect of age was revealed between all three groups. *Post hoc* pairwise comparisons illustrated the though mean age of the Sport students was lower than the mean age of the Music and Other group of students, the difference was not significant once adjusted for multiple comparisons level (*p* = 0.006 and *p* = 0.004, respectively). However, when comparing Music and Sport groups only, the difference between Age in these groups was significant (*p* < 0.001, *Mean Difference* = 1.33, *CI* = 0.61–2.06).

**TABLE 4:** Results between all three student groups, and between performance-orientated (music and sport) students.

Variable	Between three groups	Missing data (*n*)	Between music and sport	Missing data (*n*)
Age	*H* = 23.98, *df* = 2, *p* < 0.001	0	*t*_(186.80)_ = 3.61, *p* < 0.001, *d* = 0.51	0
Gender	*ns* (*p* = 0.06)	0	*ns* (*p* = 0.07)	0
Level of education	*H* = 11.22, *df* = 2, *p* = 0.004	0	*t*_(183.15)_ = 3.33, *p* = 0.001, *d* = 0.49	0
Current physical injury	*H* = 12.10, *df* = 2, *p* = 0.002	0	*ns* (*p* > 0.1)	0
Current prescription medication	*ns* (*p* > 0.2)	0	*ns* (*p* = 0.09)	0
Type of parental support	*ns* (*p* = 0.03)	0	*ns* (*p* > 0.3)	0
Perceived competence scale	*ns* (*p* = 0.03)	0	*ns* (*p* = 0.02)	0
**Psychological wellbeing scale**				0
Autonomy	*ns* (*p* > 0.5)	0	*ns* (*p* > 0.9)	
Environmental mastery	*ns* (*p* > 0.6)	0	*ns* (*p* > 0.4)	
Personal growth	*ns* (*p* = 0.05)	0	*ns* (*p* = 0.05)	
Positive relations with others	*ns* (*p* = 0.05)	0	*ns* (*p* > 0.4)	
Purpose in life	*ns* (*p* > 0.6)	0	*ns* (*p* > 0.7)	
Self-acceptance	*ns* (*p* > 0.3)	0	*ns* (*p* > 0.7)	
**Revised life orientation test**		0		0
Optimism	*ns* (*p* = 0.03)		*ns* (*p* > 0.2)	
Pessimism	*ns* (*p* = 0.13)		*ns* (*p* = 0.01)	
Warwick–Edinburgh Wellbeing Scale (WEMWBS)	*ns* (*p* > 0.6)	0	*ns* (*p* > 0.5)	0
Personal Health Questionnaire (PHQ9)	*ns* (*p* > 0.9)	5	*ns* (*p* > 0.4)	4
World Health Organization Wellbeing (WHO-5)	*ns* (*p* > 0.2)	8	*ns* (*p* > 0.3)	7
**SF12**		18		17
Physical health	*ns* (*p* > 0.5)		*ns* (*p* > 0.8)	
Mental health	*ns* (*p* > 0.5)		*ns* (*p* > 0.7)	
**Ten item personality inventory (TIPI)**		15		11
Extraversion	*ns* (*p* > 0.4)		*ns* (*p* > 0.4)	
Agreeableness	*ns* (*p* > 0.8)		*ns* (*p* > 0.9)	
Conscientiousness	*ns* (*p* > 0.8)		*ns* (*p* > 0.3)	
Openness to experience	*F*_(2,256)_ = 6.518, *p* = 0.002, $\eta_{\text{p}}^{2}$ = 0.049		*t*_(188)_ = 2.97, *p* = 0.003, *d* = 0.46	
Emotional stability	*ns* (*p* > 0.3)		*ns* (*p* > 0.1)	
Kessler psychological distress (K10)	*ns* (*p* > 0.6)	16	*ns* (*p* > 0.5)	12

Although differences were apparent in the level of education between all three groups (in terms of the frequency of undergraduate and postgraduate students), the differences did not withstand adjustment for multiple comparisons between the three groups. However, the difference was significant between Music and Sport only (*p* < 0.001, *Mean Difference* = 0.221, *CI* = 0.09–0.35). There were significantly more postgraduate students in music compared to sport, with a medium to large effect size (see Table 4).

A significant difference between all three groups was revealed for course related physical injury (four sport students, two music students, but no other students suffered these issues). *Post hoc* pairwise comparisons showed that the difference was driven by the Sport and Other student groups (*p* = 0.001, Music/Other, *p* = 0.031). Confirming this, there was no significant difference between groups when comparing Music and Sport students only.

**FIGURE 1:**
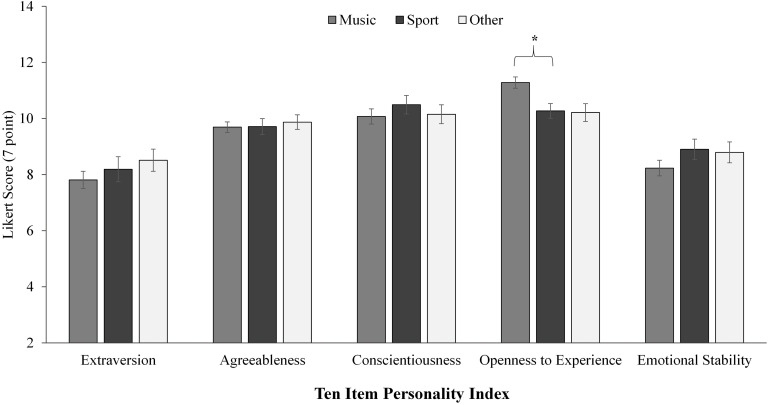
Results for between group analysis of the 10 item personality inventory (TIPI). Significance level *p* < 0.003, denoted *. Error bars represent standard error. TIPI is scored on a 7 point Likert scale, factors are scored summatively (two items each factor).

Significant differences were also revealed between all three groups for the TIPI personality factor of Openness to Experience. *Post hoc* pairwise comparisons showed music students score higher than sport (*p* = 0.002), but not than other students once adjusted for multiple comparisons (*p* = 0.004), as illustrated in Figure 1.

When comparing only music and sport student groups for Openness to Experience, music students scored significantly higher than sport students (*p* = 0.003, *Mean Difference* = 1.01, *CI* = 0.34–1.69) with a medium to large effect size (see Table 4).

### Inferential Statistical Results – Within Groups

**FIGURE 2:**
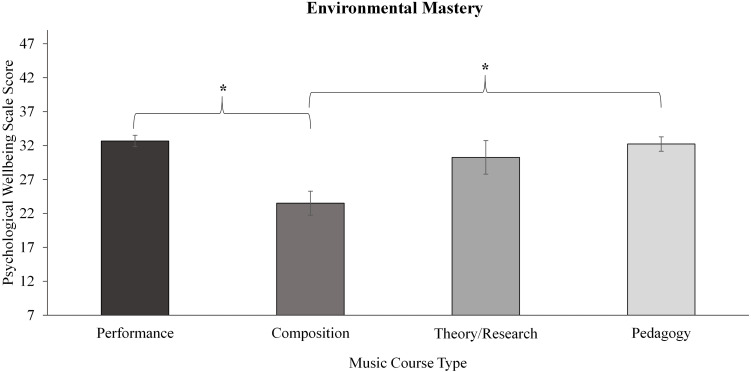
Within music course type differences for the environmental mastery factor of the psychological wellbeing scale. Significance level denoted as: *p < 0.005. Error bars represent standard error.

**FIGURE 3:**
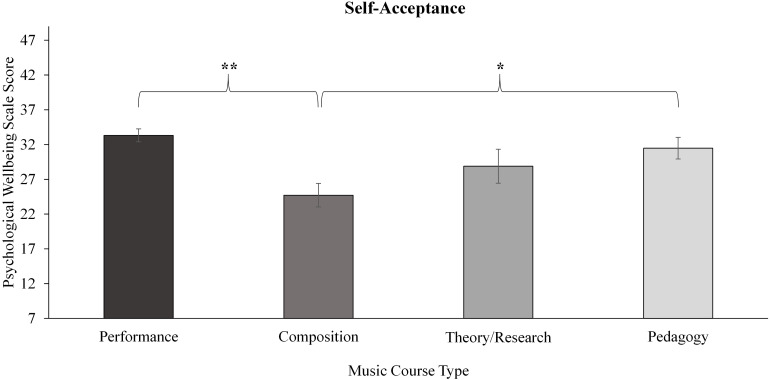
Within music course type differences for the self-acceptance factor of the psychological wellbeing scale. Significance levels denoted as: **p < 0.001, *p < 0.01. Error bars represent standard error.

To explore potential within-group differences for the performance-orientated student groups, within-group analyses for the music and sport student groups were conducted by course and performance type, the results of which are illustrated in Table 5. Significant effects of course and performance type were revealed for music students only. Of the six factors of wellbeing in the PWB scale, two differed significantly according to course type for the music students, as depicted in Figures 2, 3.

**TABLE 5:** Results of within-group analyses for music and sport student groups according to course and performance type.

Variable	Music		Sport	
	Course type	Performance type	Course type	Performance type
Current physical injury	*ns* (*p* > 0.3)	*ns* (*p* > 0.7)	*ns* (*p* > 0.9)	*ns* (*p* > 0.4)
Current prescription medication	*ns* (*p* > 0.6)	*ns* (*p* = 0.02)	*ns* (*p* > 0.4)	*ns* (*p* > 0.7)
Type of parental support	*ns* (*p* > 0.5)	*ns* (*p* > 0.7)	*ns* (*p* > 0.8)	*ns* (*p* > 0.5)
Perceived competence scale	*ns* (*p* = 0.1)	*ns* (*p* = 0.09)	*ns* (*p* > 0.04)	*ns* (*p* > 0.8)
**Psychological wellbeing scale**				
Autonomy	*ns* (*p* > 0.2)	*ns* (*p* > 0.5)	*ns* (*p* > 0.1)	*ns* (*p* > 0.4)
Environmental mastery	*H* = 17.29, *df* = 3, *p* = 0.001	*ns* (*p* > 0.2)	*ns* (*p* > 0.1)	*ns* (*p* > 0.9)
Personal growth	*ns* (*p* = 0.023)	*ns* (*p* = 0.004)	*ns* (*p* > 0.2)	*ns* (*p* > 0.8)
Positive relations with others	*ns* (*p* = 0.004)	*ns* (*p* > 0.3)	*ns* (*p* = 0.02)	*ns* (*p* > 0.6)
Purpose in life	*ns* (*p* = 0.004)	*ns* (*p* > *0.08*)	*ns* (*p* > 0.5)	*ns* (*p* > 0.6)
Self-acceptance	*H* = 15.79, *df* = 3, *p* = 0.001	*ns* (*p* > 0.3)	*ns* (*p* > 0.1)	*ns* (*p* > 0.3)
**Revised life orientation test**				
Optimism	*ns* (*p* = 0.014)	*ns* (*p* > 0.7)	*ns* (*p* > 0.2)	*ns* (*p* > 0.7)
Pessimism	*ns* (*p* > 0.1)	*ns* (*p* > 0.1)	*ns* (*p* > 0.6)	*ns* (*p* > 0.3)
WEMWBS	*H* = 13.60, *df* = 3, *p* = 0.003	*ns*, (*p* < 0.2)	*ns* (*p* > 0.1)	*ns* (*p* > 0.5)
PHQ 9	*ns* (*p* = 0.01)	*ns* (*p* > 0.9)	*ns* (*p* > 0.1)	*ns* (*p* > 0.9)
WHO-5	*H* = 12.15, *df* = 3, *p* = 0.007	*ns* (*p* < 0.8)	*ns* (*p* > 0.2)	*ns* (*p* > 0.6)
SF12 physical health	*ns* (*p* > 0.1)	*ns* (*p* > 0.9)	*ns* (*p* > 0.4)	*ns* (*p* > 0.9)
SF12 mental health	*ns* (*p* = 0.04)	*ns* (*p* > 0.6)	*ns* (*p* > 0.5)	*ns* (*p* > 0.9)
**TIPI**				
Extraversion	*ns* (*p* = 0.1)	*ns* (*p* = 0.08)	*ns* (*p* > 0.5)	*ns* (*p* > 0.7)
Agreeableness	*ns* (*p* > 0.3)	*ns* (*p* > 0.8)	*ns* (*p* > 0.6)	*ns* (*p* > 0.4)
Conscientiousness	*ns* (*p* = 0.007)	*ns* (*p* > 0.3)	*ns* (*p* > 0.8)	*ns* (*p* > 0.7)
Openness to experience	*ns* (*p* > 0.4)	*H* = 15.07, *df* = 3, *p* = 0.002	*ns* (*p* = 0.09)	*ns* (*p* > 0.8)
Emotional stability	*ns* (*p* > 0.1)	*ns* (*p* > 0.3)	*ns* (*p* > 0.2)	*ns* (*p* > 0.8)
Kessler psychological distress (K10)	*ns* (*p* = 0.08)	*ns* (*p* > 0.9)	*ns* (*p* > 0.4)	*ns* (*p* > 0.9)

**Statistical significance level (adjusted for multiple comparisons according to Bonferroni method) was p < 0.003. Non-significant results (ns) are provided in brackets for full reporting*.*

#### Environmental Mastery

A significant effect of music course type was revealed (Figure 2). *Post hoc* pairwise comparisons showed significant differences whereby the Composition group scored less than Performance with small to medium effect sizes (*p* < 0.001, *ES r* = 0.36) and Pedagogy (*p* = 0.001, *ES r* = 0.28) and to a lesser extent (i.e., did not reach adjusted level for statistical significance), Theory/Research (*p* = 0.015).

#### Self-Acceptance

A significant effect of music course type was revealed (Figure 3). *Post hoc* pairwise comparisons showed significant differences whereby the Composition group scored significantly less than Performance with a medium effect size (*p* < 0.001, *ES r* = 0.33) and to a lesser extent, Pedagogy (*p* = 0.008).

**FIGURE 4:**
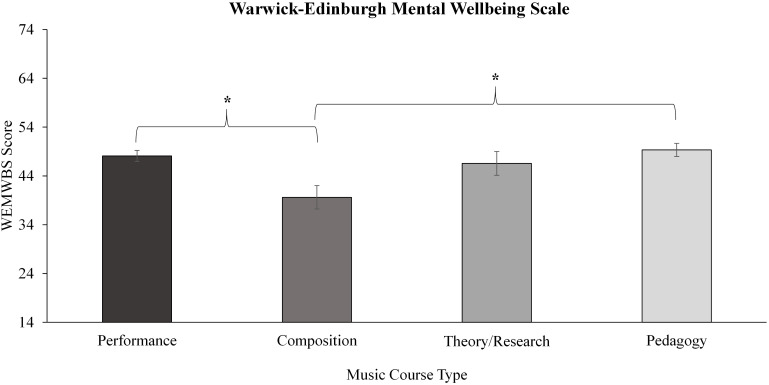
Within music course type differences for the Warwick–Edinburgh Wellbeing Scale. Significance level denoted as: *p < 0.005. Error bars represent standard error.

A significant effect of music course type was revealed in the WEMWBS measure of wellbeing (Figure 4). *Post hoc* pairwise comparisons showed significant differences for the WEMWBS whereby the Composition group scored significantly lower than Performance (*p* < 0.001, *ES r* = 0.30), and compared to Pedagogy (*p* = 0.001, *ES r* = 0.29) with medium effect sizes, but not compared to Theory/Research (*p* = 0.03).

**FIGURE 5:**
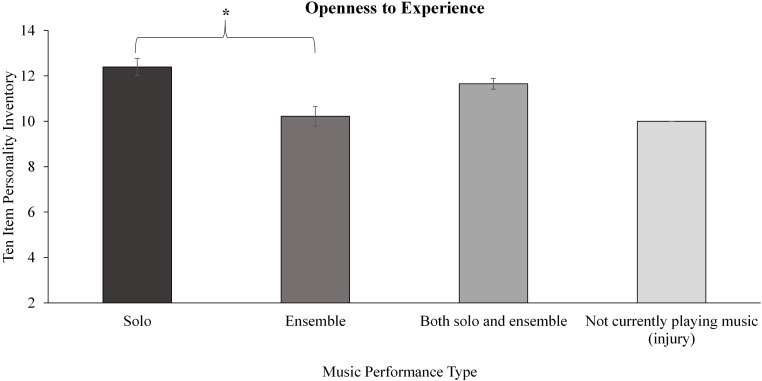
Within music performance type differences for the TIPI personality factor, openness to experience. Significance level denoted as: *p < 0.001. Error bars represent standard error.

For the TIPI, a significant effect of music performance type was revealed for the openness to experience factor. Pairwise comparisons showed significant differences whereby the soloists scored significantly higher than ensemble players (*p* < 0.001, *ES r* = 0.31) with a medium effect size, as depicted in Figure 5.

### Correlations Between Measures

As shown in Table 6, for the whole sample, within wellbeing measures, the WHO-5 and the WEMWBS were highly positively correlated. Positive correlations were also found between both the WHO-5 and the WEMWBS scores and the six PWB factors. Health and wellbeing measures were not systematically associated. The WHO-5, WEMWBS and all six items of PWB were moderately to strongly negatively correlated with both K10 and PHQ9 scores. The K10 and PHQ9 were highly positively correlated. The SF12 physical and mental health components were weakly negatively correlated. However, the SF12 mental health was not associated with the K10, PHQ9, or any of the wellbeing measures.

**TABLE 6:** Results of Pearson correlation analyses (two-tailed) between wellbeing, health and trait measures for whole sample.

	1	2	3	4	5	6	7	8	9	10	11	12	13	14	15	16	17	18	19	20
(1) WHO-5	–																			
(2) WEMWBS	0.79*	–																		
(3) PWB autonomy	0.26*	0.37*	–																	
(4) PWB environmental mastery	0.67*	0.71*	0.44*	–																
(5) PWB personal growth	0.35*	0.49*	0.34*	0.47*	–															
(6) PWB positive relations with others	0.48*	0.63*	0.27*	0.58*	0.50*	–														
(7) PWB purpose in life	0.45*	0.50*	0.25*	0.55*	0.48*	0.44*	–													
(8) PWB self-acceptance	0.66*	0.73*	0.39*	0.72*	0.53*	0.58*	0.52*	–												
(9) SF12 physical health	0.13	0.15	0.07	0.10	0.04	0.08	0.05	0.05	–											
(10) SF12 mental health	0.06	0.02	−0.01	−0.03	−0.11	−0.01	0.06	−0.01	−0.15	–										
(11) PHQ9	−0.69*	−0.66*	−0.21*	−0.65*	−0.25*	−0.41*	−0.47*	−0.58*	−0.11	0.07	–									
(12) K10	−0.71*	−0.70*	−0.30*	−0.69*	−0.32*	−0.41*	−0.50*	−0.62*	−0.14	0.01	0.81*	–								
(13) Perceived competence	0.34*	0.38*	0.39*	0.44*	0.41*	0.31*	0.31*	0.41*	0.08	0.03	−0.28*	−0.30*	–							
(14) LOT-R optimism	0.48*	0.52*	0.21*	0.50*	0.40*	0.35*	0.29*	0.64*	0.02	−0.04	−0.43*	−0.48*	0.25*	–						
(15) LOT-R pessimism	0.46*	0.53*	0.30*	0.49*	0.53*	0.38*	0.40*	0.65*	0.12	−0.02	−0.49*	−0.52*	0.29*	0.54*	–					
(16) TIPI extraversion	0.26*	0.34*	0.30*	0.32*	0.40*	0.43*	0.31*	0.36*	0.07	−0.05	−0.17*	−0.22*	0.22*	0.28*	0.28*	–				
(17) TIPI agreeableness	0.22*	0.27*	0.01	0.18	0.18	0.27*	0.09	0.19*	0.04	−0.01	−0.22*	−0.28*	0.05	0.31*	0.17*	0.09	–			
(18) TIPI conscientiousness	0.36*	0.34*	0.15	0.50*	0.25*	0.24*	0.46*	0.34*	0.13	0.02	−0.38*	−0.33*	0.25*	0.18*	0.22*	0.02	0.13	–		
(19) TIPI openness to experience	0.33*	0.40*	0.28*	0.26*	0.48*	0.33*	0.23*	0.28*	0.12	−0.14	−0.17*	−0.24*	0.27*	0.29*	0.31*	0.34*	0.17*	0.11	–	
(20) TIPI emotional stability	0.55*	0.54*	0.36*	0.55*	0.28*	0.34*	0.30*	0.53*	0.10	−0.03	−0.54*	−0.61*	0.28*	0.46*	0.41*	0.17*	0.24*	0.32*	0.18*	–

Significance levels denoted: * p < 0.001.

The SF12 physical health showed a weak correlation with the WHO-5 and the WEMWBS but not with any of the PWB factors. As data was gathered regarding the prevalence of current physical injury among participants, a one-tailed correlation was conducted on these data with the SF12 physical health. This showed a negative association, *r*_*(255)*_ = −0.19, *p* = 0.001, suggesting the existence of physical injury was associated with lower SF12 scores as would be expected.

Regarding the traits, Perceived Competence, Optimism and Pessimism correlated positively with each other, and with all the wellbeing measures, but negatively with K10 and PHQ9, and not at all with the SF12 factors. For the TIPI, the WHO-5 and WEMWBS correlated positively and strongly with all five personality traits. From the PWB, most factors were correlated positively and strongly with all five personality traits. However, Autonomy was not associated with Agreeableness and only weakly with Conscientiousness, and Purpose in Life was not associated with Agreeableness. Optimism and pessimism were positively associated with all the TIPI traits.

### Multiple Regression Analyses for Trait Measures

Finally, multiple regression analyses were carried out to investigate how traits measures (perceived competence, life orientation [optimism/pessimism] and personality [extraversion, agreeableness, conscientiousness, openness to experience and emotional stability]) predicted health and wellbeing by group. Based on the correlation analyses, the WHO-5 was chosen as the most parsimonious outcome variable incorporating both aspects of the two-continua model^3^. Forced entry was used to create models from the predictor variables as is common in exploratory analyses (Studenmund and Cassidy, 1987). Results suggest different models for each student group as depicted in Table 7.

**TABLE 7:** Results of multiple regression using trait variable as predictors on the WHO-5 measure of wellbeing.

Group and predictors	*R*^2^	Adjusted *R*^2^	ANOVA	β*^*a*^*	β*^*b*^*	*t-*value	*p*-value
**Other students**	0.65	0.42	*F*_(2, 66)_ = 23.22, *p* < 0.001	7.95			
Optimism					0.42	3.19	*p* = 0.002
Emotional stability					0.29	2.19	*p* = 0.032
**Sport students**	0.67	0.49	*F*_(3,62)_ = 15.96, *p* < 0.001	−10.31			
Emotional stability					0.39	3.35	*p* = 0.001
Optimism					0.28	2.60	*p* = 0.012
Conscientiousness					0.22	2.05	*p* = 0.045
**Music students**	0.69	0.47	*F*_(4,127)_ = 27.63, *p* < 0.001	−15.96			
Emotional stability					0.34	4.72	*p* < 0.001
Perceived competence					0.23	3.37	*p* = 0.001
Optimism					0.26	3.63	*p* < 0.001
Conscientiousness					0.18	2.67	*p* = 0.009

**β^*a*^, unstandardized model constant, β^*b*^, standardized predictor coefficients*.*

## Discussion

Studies of music and sport students have suggested high levels of wellbeing (e.g., Malebo et al., 2007; Ascenso et al., 2018a), yet worrying levels of vocation-related physical and mental health problems (e.g., Hildebrandt et al., 2012; Bauman, 2016; Spahn et al., 2017; Schinke et al., 2018). Bearing in mind this paradox, and against a background which suggests there are generally poor levels of mental health in HE students, this study compared wellbeing and trait patterns in music, sport and other students. By doing so it investigated measures that can be combined in a parsimonious yet sensitive way to provide a multi-facetted picture of ‘health and wellbeing’ for different student groups.

### Students’ Health and Wellbeing

The results supported the general findings that students in HE self-report significantly lower than population average ratings of mental health and wellbeing (WHO-5, WEMWBS, and SF12 Mental Health), but not in terms of physical health according to the SF12 Physical health component. Although these findings should be viewed against the limitations of the gathered and published normative data (i.e., socio-economic status was not accounted for, and the SF12 is based on the general United States population), the replication within this study (i.e., the multiple measures) suggests that the mental health and wellbeing of these HE student participants was compromised. The K10 and PHQ9 suggested that approximately one third of the students suffered with moderate to severe psychological distress and/or depression. There were no significant differences between groups for these measures, suggesting a general psychological malaise for students in HE, who struggle to find a balance between eustress and distress.

We expected that music and sports students would suffer with more physical complaints compared to other students due to the embodied nature of their vocational studies. Although more music and sport students did report higher instances of currently suffering with a physical injury related to their course, compared to other students (16 and 23%, respectively, in comparison to 4% of other students), this difference did not reach the level of statistical significance. Whilst good levels of physical fitness might be expected for sports students, that is not the case for music students, as Williamon et al. (2009) reported that music students generally fall below recommended body mass indices and that under 40% of music students achieve healthy levels of cardio-vascular fitness. However, the findings herein provide some support for a recent study, which suggested an adequate amount of physical activity was undertaken by music students (Araújo et al., 2020).

### Within Groups: Homogeneity and Variety of Sport and Music Students

According to Spahn et al. (2017) multi-center studies, three categories of music students exist: those who have no problems and attribute this to luck, therefore taking no action to protect themselves (40%), those who have a healthy adaptive attitude and engage in health-promoting behaviors (40%), and a third group who need care and use medicine and health professionals to attend to their needs (20%). Although provisions were made in schools of music for health-promoting activities, even when these courses were taken, playing-related injuries persisted, increasing from 29% in the first year, to 42% in the second year, then dropping slightly to 36% in the third year of study. Students tended to engage more in physical/body-orientated courses compared to those focused on psychological preparation, suggesting a ‘play-on’ mentality persists.

Underpinning this, Vaag et al. (2014) conducted a study of the occupational stress associated with being a freelance musician. They found that being sick was associated with not being able to play, which was described by participants as an ‘impossible situation.’ The authors suggested that when considering wellbeing for musicians, there must be an understanding of the dynamic relationships between contextual demands, personal and social resources. For example, not many people receive applause just for doing their job, but can then be savaged by reviews for the very same performance. They suggest self-efficacy (cf. Bandura, 1977) is an essential personal resource of musicians, and that more than being ‘thick-skinned’ (p. 218), having a Protean career^4^ as a musician involves transactional processes including risk-taking and vulnerability, that requires shifting mindsets and a range of behavioral repertoires (Vaag et al., 2014).

In order to understand the directionality of the connection between such vocation-related physical jeopardy and mental health and wellbeing, one future goal for researchers could be to develop a specific scale for musicians, as has been devised in sport (Kouali et al., 2020). Previous research has suggested that sport participation at a collective level can create a shared identity, connectedness and a sense of belonging (Malebo et al., 2007). Kouali et al. (2020) found that a unidimensional measure adequately captured eudaimonic wellbeing for sport students. The findings herein support this perspective as no within group differences were found in measures of wellbeing for sport students. In contrast, significant differences were apparent not only in one of the global wellbeing scales (WEMWBS), but also two of the Psychological Wellbeing scale (PWB) for the music students. The pattern of findings that emerged from the PWB showed that music students enrolled on a composition/song writing (henceforth composition) course scored significantly lower than music performance students for *environmental mastery*, and for *self-acceptance*. As no differences were apparent for the PHQ9 or K10 these issues cannot be solely related to anxiety and depression alone.

Light can be shone on these findings by the qualitative research undertaken by Ascenso and Perkins (2013). Following interviews conducted with professional musicians, they identified that composers need to take care to force themselves “not be isolated by music” (p. 387), and that differences between solo and ensemble musicians need to be addressed at educational level as being a solo musician demanded more self-regulation due to the lack of available feedback. A later paper (Ascenso et al., 2017) suggested that the social structures in group music making provide intrinsic value, such as shared memories of success and the development of a group identity. Furthermore, playing together regularly helped build “a solid positive performance narrative through the years [which] allows for a validation of self-perceptions of competence” (p. 77).

Whilst these findings regarding differences in relation to what constitutes wellbeing according to music course type and music performance offer an initial insight that may be important for considering institutional support and intervention (e.g., ensuring composers receive more opportunity for social engagement), the need for replication is apparent due to the small number of participants within each group.

### ‘Performance-Orientated’ Student Wellbeing Profile

In addition to the within-group findings, the multiple regression models demonstrate that the perceived competence was specifically linked to wellbeing for music, but not for sport and other students. For musicians, it seems, it is hard to differentiate between “being a musician and the doing of music” (Ascenso et al., 2017, p. 71). This may be particularly difficult for composers, as illustrated by this quote from the same paper “There is just not a way I could work on something else because this is who I am in my essence”. Vaag et al. (2014) also reported that musicians found it difficult to “distinguish work from the rest of their lives” (p. 209). The key, as Ascenso et al. (2017) conclude, seems to be developing self-worth, or a self-concept, that is independent of musical characteristics or achievement. As one of their interviewees, a chamber musician explained, “one thing is you as a person. Another is your performance: it’s a moment – not you.”

Ascenso and Perkins (2013) also noted that all their interviewees reported issues with emotional instability. This consensus view was supported by the addition of the personality trait *emotional stability* as a predictor in the multiple regression model in music as well as sport, and for other students. Hay and Ashman (2003) suggest that for young adults, emotional stability (described as calmness and freedom from anxiety and depression) is related to self-concept through extrinsic factors such as the role and relationship with teachers, parents and peers. Along with the inclusion of the other personality trait, *conscientiousness*, (intrinsic motivation; dependable, industrious, efficient and achievement oriented) for music and sport students, this returns us to the framework of Self-Determination Theory whereby emotional stability and conscientiousness have been described as the “most valid predictors of performance outcomes” (Barrick and Mount, 2000, p. 28). The profile of music students maps onto the three factors of Self-Determination Theory; competence, relatedness and autonomy.

Self-Determination Theory has been linked to mindset in education, in that what people believe seems to shape their motivation (Dweck, 2012). Specifically, having a growth mindset means believing that one can develop and change with effort, practice and education, and seeing learning opportunities in every challenge and failures. In comparison, having a fixed mindset (entity theory) suggests that challenge and failure are risks experienced negatively, and instead people need to ‘look talented’ to prove their existing abilities, and as such their self-worth is directly tied to judgments that negatively impact their wellbeing (Dweck, 2000; Ortiz Alvarado et al., 2019).

Dispositional optimism is a mindset related to behavior and has been positively associated with health-promoting behaviors across a lifespan (Robbins et al., 1991; Steptoe and Wardle, 2001). In this study, dispositional optimism (measured using the LOT-R) predicted wellbeing for all HE students. In their study about mental toughness and coping among athletes, Nicholls et al. (2008) included the LOT-R and found the concept of mental toughness correlated significantly with optimism. Mental toughness includes aspects of self-belief, an unshakeable faith that one can control one’s destiny, can cope with the demands of training and competition with increased determination, confidence and maintain control under pressure (Jones et al., 2002). Subcomponents of mental toughness include control, commitment, challenge and confidence (Clough et al., 2002) as well as being able to ‘tolerate physical endurance for longer’ and an enhanced ability to cope with pain (Levy et al., 2006; Nicholls et al., 2008). As optimism can reportedly be learned (Seligman, 1990), future research should investigate the role of ‘mindset’ (Dweck, 2000) in relation to beliefs about ‘talent’ and work ethic (e.g., practice regimes) in students, especially in relation to potential interventions and for longitudinal studies considering student outcomes.

### Measures of Wellbeing

The present study compared two commonly applied global-score measures of wellbeing (WHO-5 and WEMWBS) with the six-factor based PWB. The WHO-5 emerged as the most parsimonious of the included measures, which still was able to capture the different dimensions of mental health and wellbeing as per the dual continua model (Hewitt, 2019). The WHO-5 could thus offer an easy to apply tool for future studies as well as HE institutions to obtain a first indication of the health and wellbeing state of students. However, the WEMWBS was designed to specifically include hedonic aspects of wellbeing. Therefore, if brevity is not of paramount importance, using the WEMWBS rather than the WHO-5 may enable investigation relating to the concept of hedonic tone (i.e., the ability to feel pleasure). This was suggested by Eysenck et al. (1982) when considering the personality traits of sports people in relation to mindset and flow states.

Based on the extant literature, pursuing these avenues of research with musicians could be an important future step forward. The PWB was significantly correlated to all health and trait measures, with the exception of the SF12. This suggests that the PWB could offer a powerful additional tool able to capture components relevant to the health and wellbeing of performance-orientated students, including the three axes of the SDT framework. Within health measures, the SF12 emerged as an anomaly, showing weak or no correlations with other health or wellbeing measures. Thus, it appears that, especially with regard to physical health, more vocation-sensitive tools are needed for music and sport in order to capture the specific nature of ‘playing-related problems’ as suggested by Spahn et al. (2017).

### Future Directions

Within the framework of Self Determination Theory, we see that according to the measures of wellbeing used herein, while students in general do not differ in terms of perceived autonomy, for music students, perceived competence additionally predicted wellbeing outcomes, and for composition students in particular, further support will be required in terms of promoting students’ awareness of their strengths and weaknesses and their feeling of mastery over one’s environment. Awareness of these needs may inform health and wellbeing promotion strategies in music schools. Institutions could, for instance, aim at providing increased positive and structured feedback in social settings for composition students to support their wellbeing.

As with all cross-sectional studies, the findings herein do not help us understand the development of adaptive and maladaptive behaviors in relation to general HE stressors as well as particular stressors associated with expert performance vocation-based courses. Furthermore, the assumption that students choosing vocational courses directly related to long-term interests and practice (i.e., music and sport practice developing into research applications in HE) was not evaluated in this study. Future studies should take care to include a direct assessment of long-term participation in vocational activities prior to HE. This also relates to the characterization of ‘control groups’ so as to capture potential differences between students who progress to later periods of their studies and those who drop out, effectively self-selecting. Similarly, care must be taken to consider the impact and specificity of playing-related injuries and other issues (such as performance anxiety) not only for music and sports student, but for all students as these issues may negatively affect course participation and exam performance for example. Furthermore, the sample for this study was mainly European in nature. Whilst school systems do differ across Europe, the Bologna Process has harmonized HE. Therefore, future studies may wish to consider cultural differences from a world-wide perspective.

Overall, from the perspective that HE institutions have a higher-purpose for society than providing education for some (Chickering and Reisser, 1993), longitudinal research is required to understand how the nature of eustress and distress changes throughout the HE period and which approaches are of benefit (or form obstacles) to which students (e.g., as shown in intervention studies such as Malebo et al., 2007; Steyn et al., 2016). This study therefore included multiple measures of state and trait concepts in order to investigate what level of assessment is required to understand the nature of the students in general and in terms of the specific needs of, in this instance, performance-orientated students with specialist expertise. This approach yielded findings that provide initial insights, especially in relation to the health and wellbeing of music students, but it also provides a potential model for research that can be adapted to other avenues of investigation.

## Conclusion

We surveyed music, sport and other students to compare them on selected wellbeing, health, and traits measures. As expected, all students reported increased health and wellbeing issues compared to the general population. However, distinct profiles of wellbeing emerged for ‘performance-orientated’ students, and for sub-groups of students within musicians, suggesting the need for vocation-specific and course-tailored wellbeing agendas and health promotion strategies for music and sport institutions. Results highlighted the interconnection between individual traits – like perceived competence, emotional stability, conscientiousness, and dispositional optimism – and wellbeing in students. These findings resonate with the Self Determination Theory and suggest the need for institutions to embed health and wellbeing into a ‘living curriculum’, driving the whole institution approach to overcome stigma associated with support systems. Among the wellbeing measures applied, the WHO-5 emerged as a parsimonious yet sensitive tool to be used in future studies, e.g., to evaluate the effectiveness of interventions. The PWB was able to capture different components of wellbeing and could offer an important additional tool in future investigations. However, future studies should also consider including trait measures to disentangle underlying mechanisms affecting the different wellbeing components.

## Data Availability Statement

The raw data supporting the conclusions of this article will be made available by the authors upon request, without undue reservation.

## Ethics Statement

The studies involving human participants were reviewed and approved by Ethics Committees of Cardiff Metropolitan University/Prifysgol Metropolitan Caerdydd, United Kingdom. The patients/participants provided their written informed consent to participate in this study.

## Author Contributions

EA and DR designed the study and collected the data. DR analyzed the data. EA and DR wrote the manuscript. DW was responsible for the ethical approval. All authors reviewed and edited the manuscript and approved the final version.

## Conflict of Interest

The authors declare that the research was conducted in the absence of any commercial or financial relationships that could be construed as a potential conflict of interest.

## Footnotes

1 https://www.orthotoolkit.com/sf-12/

2 www.qualtrics.com

3 A review of studies using WHO-5 describes it as a sensitive and specific screening tool for depression (Topp et al., 2015).

4 A career in which the individual, not an organization is in charge (Hall, 2004).
