# Supplementary material for: Health and Wellbeing in Higher Education: A Comparison of Music and Sport Students Through the Framework of Self Determination Theory
Source: Front Psychol. 2020 Oct 28;11:566307. doi: 10.3389/fpsyg.2020.566307 (PMC7655782; doi:10.3389/fpsyg.2020.566307)
Supplement: Supplementary file 1 [file Data_Sheet_1.PDF]

Supplementary Material A – Student Wellbeing Survey

Welcome to our survey about Student Wellbeing.

In this study, we would like to compare the impact different study courses and/or vocations have on the physical and mental health of students.
Students' wellbeing is an important and still under-researched topic. Your answers will help us better understand what wellbeing means for students and develop strategies to promote wellbeing throughout their studies.

We would like to assure you that all your answers are collected anonymously for the purpose of research only. Please click on the link for the full participant information sheet: Participant Information Sheet

This study has been granted ethical approval by the Ethics Committees of Cardiff Metropolitan University/Prifysgol Metropolitan Caerdydd in the U.K.

It takes 15-25 minutes to complete this survey. We very much appreciate the time you take to answer the questions. Therefore, for each properly completed survey we will **donate £2** to www.studentminds.org.uk a charity set up especially to provide help for students. It is based in the UK but provides lots of great online resources for students worldwide. During the survey, we will also give you some feedback (based on your answers) on your personality profile. This will include these five factors: Agreeableness, Conscientiousness, Emotional Stability, Extraversion, Openness to Experience.

If you have any questions please contact the researchers:
Dr. Dawn Rose dawn.rose@hslu.ch
Dr. David Wasley dwasley@cardiffmet.ac.uk
Dr. Elena Alessandri elena.alessandri@hslu.ch

This survey will take 15-25 minutes to complete.
You can quit the survey at any time if you no longer want to participate.

Please confirm the following:

I understand that my participation is voluntary and that I am free to withdraw at any time without giving any reason. I give permission to the researchers to have access to my anonymous responses and I understand that my name will not be linked with the research materials and I will not be identifiable in the reports that result from the study.
I confirm that I am 18 years or older AND a student in higher education.

- ○ Yes, I agree to participate
- ○ No, I do not wish to participate

Please verify that you speak and understand English?

- ○ Yes
- ○ No

Thanks!

To start, we would like to know more about you.
Please choose the description of gender you prefer for yourself.

- ○ Male
- ○ Female
- ○ Another description ____

Please enter your age in years.

____

In which country do you currently reside?

____

Please choose a term that best describes your current level of education.

- ○ Undergraduate / Bachelor Course (e.g., BA, BA(Hons), BSc)
- ○ Master Course (e.g., MA, MSc, MMus)
- ○ Alternative Professional Qualification (e.g., Diploma, Certificate, Laureate)
- ○ Doctoral (e.g., PhD, DMus, Ed.D.)

Please write the name of your course of study in the box below.

____

Does your course of study focus on learning, doing or playing **MUSIC**?

- ○ Yes
- ○ No

Does your course of study focus on learning, doing or playing **SPORTS**?

- ○ Yes
- ○ No

As a music student, does your course focus more on...

- ○ Performance
- ○ Composition / Song Writing
- ○ Theory / Research
- ○ Pedagogy / Teaching & Learning

As a music performance student, would you mainly describe yourself as a...

- ○ Soloist
- ○ Band / Ensemble / Orchestra Player
- ○ I do both solo and group music playing equally
- ○ I don't currently practice or play music.

As a sports student, does your training mainly involve...

- ○ Training, Competing & Coaching
- ○ Sports Science & Research
- ○ Sports Related Health and Remediation
- ○ Sports Related Teaching, Learning and/or Management

As a sports student, do you mainly train for...

- ○ Individual sports (e.g., tennis, track & field, swimming, weight lifting)
- ○ Team sports (e.g., football, volleyball, hockey, curling)
- ○ My sport involves both individual and team aspects (e.g., sprint and relay)
- ○ I don't currently practice or play sports.

Are you currently suffering from a **physical issue or difficulties** related to your chosen area of study?
For example, sports people can get injured, musicians can suffer hearing damage.

- ○ Yes
- ○ No

Please could you provide further detail about the physical issue or difficulty you are currently facing and how it relates to your course?

____

____

____

Are you currently taking any prescribed medication?

- ○ Yes
- ○ No

Please describe the medication and what it was prescribed for?

____

Now we would like to ask you how you **feel** about your course of study specifically.
Not at all true – Not true – Slightly true – Moderately true – Mostly true – True – Very true

- I feel confident in my ability to learn the material in my course
- I am capable of learning the material in my course
- I am able to achieve my goals in my course
- I feel able to meet the challenge of performing well in my course

How would you describe the support you have received from your family for your chosen course?

- ○ Supportive / Positive
- ○ Neutral
- ○ Conflicting / Negative

Next we would like to ask some questions about your **current state of wellbeing** (i.e., how you are feeling right now). Please click on one response next to each statement to indicate how much you agree or disagree with that statement. Try not to overthink how you answer.
<u>Remember:</u> This is about how you feel **right now**. There are no right or wrong answers.
Strongly agree – Agree – Somewhat agree – Neither agree nor disagree – Somewhat disagree – Disagree – Strongly disagree

- For me, life has been a continuous process of learning, changing, and growth.
- In general, I feel I am in charge of the situation in which I live.
- People would describe me as a giving person, willing to share my time with others.
- I am not interested in activities that will expand my horizons.
- I enjoy making plans for the future and working to make them a reality.
- Most people see me as loving and affectionate.
- In many ways I feel disappointed about my achievements in life.
- I live life one day at a time and don't really think about the future.
- I tend to worry about what other people think of me.
- When I look at the story of my life, I am pleased with how things have turned out.
- I have difficulty arranging my life in a way that is satisfying to me.
- My decisions are not usually influenced by what everyone else is doing.
- I gave up trying to make big improvements or changes in my life a long time ago.
- The demands of everyday life often get me down.
- I have not experienced many warm and trusting relationships with others.
- I think it is important to have new experiences that challenge how you think about yourself and the world.
- Maintaining close relationships has been difficult and frustrating for me

- My attitude about myself is probably not as positive as most people feel about themselves.
- I have a sense of direction and purpose in life.
- I judge myself by what I think is important, not by the values of what others think is important.

Thanks, you are helping us understand much more about wellbeing for students.
Next, we would like to ask you about your **outlook on life**. Remember: There are no "correct" or "incorrect" answers, but please be as honest and accurate as possible.
Strongly disagree – Disagree – Neutral – Agree – Strongly agree

- In uncertain times, I usually expect the best.
- If something can go wrong for me, it will.
- I'm always optimistic about my future.
- I hardly ever expect things to go my way.
- I rarely count on good things happening to me.
- Overall, I expect more good things to happen to me than bad.

Here are some further questions on your **current state of wellbeing**, how you feel **right now**. Please click on one response next to each statement to indicate how much you agree or disagree with that statement. Try not to overthink how you answer.
Strongly agree – Somewhat agree – A little agree – Neither agree nor disagree – A little disagree – Somewhat disagree – Strongly disagree

- In general, I feel confident and positive about myself.
- I have been able to build a living environment and a lifestyle for myself that is much to my liking.
- I tend to be influenced by people with strong opinions.
- I do not enjoy being in new situations that require me to change my old familiar ways of doing things.
- I do not fit very well with the people and the community around me.
- I know that I can trust my friends, and they know they can trust me.
- When I think about it, I haven't really improved much as a person over the years.
- Some people wander aimlessly through life, but I am not one of them.
- I often feel lonely because I have few close friends with whom to share my concerns.
- When I compare myself to friends and acquaintances, it makes me feel good about who I am.
- I don't have a good sense of what it is I'm trying to accomplish in life.
- I sometimes feel as if I've done all there is to do in life.
- I feel like many of the people I know have gotten more out of life than I have.
- I have confidence in my opinions, even if they are contrary to the general consensus.
- I am quite good at managing the many responsibilities of my daily life.
- I have the sense that I have developed a lot as a person over time.

- I enjoy personal and mutual conversations with family members and friends.
- My daily activities often seem trivial and unimportant to me.
- I like most parts of my personality.
- It's difficult for me to voice my own opinions on controversial matters.
- I often feel overwhelmed by my responsibilities.

Thank you for continuing to complete our questionnaire. Next we would like to ask you about your **feelings and thoughts** over the **last two weeks.** Please click the answer that best describes your experience during this time...
None of the time – Rarely – Some of the time – Often – All of the time

- I've been feeling optimistic about the future.
- I've been feeling useful.
- I've been feeling relaxed.
- I've been feeling interested in other people.
- I've had energy to spare.
- I've been dealing with problems well.
- I've been thinking clearly.
- I've been feeling good about myself.
- I've been feeling close to other people.
- I've been feeling confident.
- I've been able to make up my own mind about things.
- I've been feeling loved.
- I've been interested in new things.
- I've been feeling cheerful.

Over the **last two weeks**, how often have you been bothered by any of the following problems?
Not at all – Several days – More than half the days – Nearly everyday

- Little interest or pleasure in doing things?
- Feeling down, depressed, or hopeless?
- Trouble falling or staying asleep, or sleeping too much?
- Feeling tired or having little energy?
- Poor appetite or overeating?
- Feeling bad about yourself - or that you are a failure or have let yourself or your family down?
- Trouble concentrating on things, such as reading the newspaper or watching television?

- Moving or speaking so slowly that other people could have noticed? Or the opposite - being so fidgety or restless that you have been moving around a lot more than usual?
- Thoughts that you would be better off dead or of hurting yourself in some way?

The following statements are also about how you have **felt in general** over the **last two weeks.**
All of the time – Most of the time – More than half of the time – Less than half of the time – Some of the time – At no time

- I have felt cheerful and in good spirits.
- I have felt calm and relaxed.
- I have felt active and vigorous.
- I woke up feeling fresh and rested.
- My daily life has been filled with things that interest me.

Now we would like to know more about your **health** over the **last four weeks**.
In general, over the last four weeks would you say your health has been...

○ Excellent

○ Very Good

○ Good

○ Fair

○ Poor

During the **last four weeks**, has your **health limited you** in the following activities that people might do in a typical day?
Yes, limited a lot – Yes, limited a little – No, not limited at all

- **Moderate activities**, such as moving a table, pushing a vacuum cleaner, bowling, or playing golf?
- Climbing **several** flights of stairs?

The following two questions are about your **physical health** and your daily activities during the **last four weeks**.
Yes – No

- Have you **accomplished less** than you would like as a result of your physical health?
- Were you limited in the **kind of** work or other regular daily activities you do as a result of your physical health?

These questions are about your **emotions** and your daily activities during the **last four weeks**.
Yes – No

- Have you **accomplished less** than you would like as a result of any **emotional problems**, such as feeling depressed or anxious?
- Did you **not** do work or other regular activities as **carefully as usual** as a result of any **emotional problems**, such as feeling depressed or anxious?

During the **last four weeks**, how much did **pain** interfere with your normal work, including both work outside the home and housework?

- ○ Not at all
- ○ A little bit
- ○ Moderately
- ○ Quite a lot
- ○ Extremely

During the **last four weeks**, how much of the time has your **physical health or emotional problems** interfered with your social activities like visiting friends or relatives?

- ○ All of the time
- ○ Most of the time
- ○ Some of the time
- ○ A little of the time
- ○ None of the time

The next questions are about how you feel and how things have been with you during the last four weeks.
Please give one answer that comes closest to the way you have been feeling. How much of the time during the past four weeks...
All of the time – Most of the time – A good bit of the time – Some of the time – A little of the time – None of the time

- Have you felt calm and peaceful? (1)
- Did you have a lot of energy? (2)
- Have you felt downhearted and blue? (3)

The following questions are from the **Ten Item Personality Inventory** (TIPI; Gosling, Rentfrow, & Swann, 2003), an internationally standardised way of understanding some different aspects of how we are as people. The questions provide a profile of five different domains of personality.

At the end of the survey, we will show you your personality scores in comparison to the average population scores for each factor, and some information about what the current state of research suggests about these personality domains.

Please click on the button next to each statement to indicate to which extent you agree or disagree with that statement. You should rate the extent to which the pair of words applies to you, even if one description applies more strongly than the other. I see myself as...
Disagree strongly – Disagree moderately – Disagree a little – Agree a little – Agree moderately – Agree strongly

- Extroverted, enthusiastic. (1)
- Critical, quarrelsome. (2)
- Dependable, self-disciplined. (3)
- Anxious, easily upset. (4)
- Open to new experiences, complex. (5)
- Reserved, quiet. (6)
- Sympathetic, warm. (7)
- Disorganized, careless. (8)
- Calm, emotionally stable. (9)
- Conventional, uncreative. (10)

The following questions are about how you have been feeling **during the last four weeks.**
Please select the answer that best describes how you have been feeling during this time.
None of the time – A little of the time – Some of the time – Most of the time – All of the time

- About how often did you feel tired out for no good reason? (1)
- About how often did you feel nervous? (2)
- About how often did you feel so nervous that nothing could calm you down? (3)
- About how often did you feel hopeless? (4)
- About how often did you feel restless, or fidgety? (5)
- About how often did you feel so restless you could not sit still? (6)
- About how often did you feel depressed? (7)
- About how often did you feel that everything was an effort? (8)
- About how often did you feel so sad that nothing could cheer you up? (9)
- About how often did you feel worthless? (10)

All done! Here are your scores for each of the five personality domains compared to population 'norms':

For **Extraversion**, you scored **{Personal Score}** and the population average is **4.44.**
Scoring highly on this factor of personality suggests you are enthusiastic, talkative, bold, active, assertive, adventurous, NOT inactive, timid, unassertive, silent, introverted.
Studies suggest higher levels of Extraversion are associated with success in leadership and management positions, and also jobs that include sales.

For **Agreeableness**, you scored **{Personal Score}** and the population average is **5.23.**
Scoring highly on this factor of personality suggests you are warm, kind, trusting, generous, cooperative, unselfish, NOT cold, unkind, uncooperative, selfish, distrustful, disagreeable, or mean.
Studies suggest higher levels of Agreeableness are associated with success in team work, as well as altruistic tendencies such as helping others and charitable works.

For **Conscientiousness**, you scored **{Personal Score}** and the population average is **5.40.**
Scoring highly on this factor of personality suggests you are organised, dependable, hard-working, responsible, self-disciplined, practical, thorough, and thrifty, NOT careless, lazy, extravagant, impractical, negligent, or irresponsible. Studies suggest the well-structured life observed in people with higher levels of Conscientiousness is conducive to achieving performance targets, good group work, better health outcomes and longevity.

For **Emotional Stability**, you scored **{Personal Score}** and the population average is **4.83.**
Scoring highly on this factor of personality suggests you are calm, secure, relaxed, self-confident, contented, and positively motivated NOT anxious, angry, tense, nervous, discontented, unstable, easily demotivated or easily upset. Research shows people with high scores of emotional stability are confident in their own abilities and pay less attention to errors (i.e., not afraid of making mistakes) which can encourage and motivate lifelong learning and predicts job satisfaction.

For **Openness to Experience**, you scored **{Personal Score}** the population average is **5.38.**
Scoring highly on this factor of personality suggests you are imaginative, curious, reflective, analytical, sophisticated, open-minded, NOT conventional, dull or unsophisticated.
Studies suggest high scores on this factor of personality are associated with creativity, being independently-minded, liberal and insightful, as well as being happy and enjoying a good quality of life.

Thank you for completing our survey. We appreciate it took some of your valuable time.

Therefore, **for every completed survey our institution will donate £2 to the www.studentminds.org charity.**
We are hoping to recruit 600 volunteers to take part in the survey, which means you will have contributed to a donation of £1200.

We also want to help you with your studies by providing you with a chance to **win a Lenovo Yoga 2 in 1 laptop/tablet**, so if you would like to be entered into the prize draw, please also leave a contact email in the box below.

We want to thank you once again for your time and help, and leave you with these quick final questions.
Yes please – No thanks

- I would like to take part in further research about wellbeing.
- I would like you to contact me about the results of this survey.
- I would like to be entered into the PRIZE DRAW for a chance to win the Lenovo Yoga 2 in 1 laptop/tablet.

Here is my contact email address, which I authorise you to use for the purposes described above only:
____

We have used six different standardised measures of general wellbeing to try to understand which measures best reflect the specific needs and priorities of students. We also looked at some traits that have been proposed to support wellbeing, such as optimism/pessimism, personality, and emotional intelligence, as well as your own beliefs about your ability to undertake your course.

Please do remember to check out **www.StudentMinds.org.uk** for their excellent resources regarding wellbeing for students. Thank you once again for your time and contribution to the research.

Best regards,
Dr. Dawn Rose & Dr. Elena Alessandri,
Department of Music, Lucerne University of Applied Sciences & Arts.
Dr. David Wasley,
Cardiff School of Sport and Health Sciences, Cardiff Metropolitan University/Prifysgol Metropolitan Caerdyadd.
