# Supplementary material for: Health and Wellbeing in Higher Education: A Comparison of Music and Sport Students Through the Framework of Self Determination Theory
Source: Front Psychol. 2020 Oct 28;11:566307. doi: 10.3389/fpsyg.2020.566307 (PMC7655782; doi:10.3389/fpsyg.2020.566307)
Supplement: Supplementary file 2 [file Data_Sheet_2.PDF]

## Supplementary Material B – Means and standard deviations of all variables for whole sample and by group

| | Whole Sample | | | Music Student Group | | | Sport Student Group | | | Other Student Group | | |
|---|---|---|---|---|---|---|---|---|---|---|---|---|
| | *N* | *Mean* | *Standard Deviation* | *n* | *Mean* | *Standard Deviation* | *n* | *Mean* | *Standard Deviation* | *n* | *Mean* | *Standard Deviation* |
| Perceived Competence Scale | 273 | 21.95 | 3.95 | 135 | 21.54 | 4.17 | 67 | 22.96 | 3.74 | 71 | 21.79 | 3.60 |
| LOT-R Optimism | 273 | 6.67 | 2.47 | 135 | 6.32 | 2.50 | 67 | 6.75 | 2.17 | 71 | 7.27 | 2.58 |
| LOT-R Pessimism | 273 | 6.98 | 2.60 | 135 | 7.19 | 2.33 | 67 | 6.13 | 2.87 | 71 | 7.38 | 2.70 |
| SF12 Physical Health | 255 | 50.27 | 9.21 | 123 | 49.98 | 9.37 | 62 | 49.65 | 9.28 | 70 | 51.33 | 8.93 |
| SF12 Mental Health | 255 | 41.40 | 13.21 | 123 | 42.18 | 13.36 | 62 | 41.44 | 13.26 | 70 | 40.00 | 12.99 |
| PWB - Autonomy | 273 | 31.29 | 6.97 | 135 | 31.15 | 7.17 | 67 | 31.04 | 6.77 | 71 | 31.80 | 6.86 |
| PWB - Environmental Mastery | 273 | 31.50 | 7.89 | 135 | 31.07 | 7.89 | 67 | 32.06 | 8.01 | 71 | 31.80 | 7.83 |
| PWB - Personal Growth | 273 | 39.56 | 5.80 | 135 | 39.81 | 5.98 | 67 | 38.04 | 6.32 | 71 | 40.52 | 4.59 |
| PWB - Positive Relations with Others | 273 | 37.10 | 7.49 | 135 | 36.93 | 7.97 | 67 | 36.00 | 6.60 | 71 | 38.46 | 7.24 |
| PWB - Purpose in Life | 273 | 35.55 | 6.58 | 135 | 35.76 | 6.67 | 67 | 35.36 | 7.49 | 71 | 35.32 | 5.46 |
| PWB - Self-acceptance | 273 | 31.87 | 8.55 | 135 | 31.28 | 8.56 | 67 | 31.69 | 8.55 | 71 | 33.17 | 8.52 |
| Level of Psychological Distress (K10) | 257 | .98 | 1.14 | 127 | 1.04 | 1.15 | 63 | 0.84 | 1.10 | 67 | 1.01 | 1.15 |
| Level of Depression (PHQ9) | 268 | 1.28 | 1.16 | 132 | 1.27 | 1.04 | 66 | 1.33 | 1.33 | 70 | 1.26 | 1.20 |
| WEMWBS | 273 | 46.84 | 9.88 | 135 | 47.03 | 9.61 | 67 | 46.04 | 10.42 | 71 | 47.24 | 9.96 |
| WHO-5 Percent | 265 | 52.41 | 20.88 | 130 | 52.74 | 20.27 | 65 | 55.82 | 22.51 | 70 | 48.63 | 20.13 |
| TIPI - Extraversion | 258 | 8.09 | 3.41 | 128 | 7.81 | 3.44 | 63 | 8.19 | 3.55 | 67 | 8.51 | 3.23 |
| TIPI - Agreeableness | 258 | 9.74 | 2.15 | 128 | 9.69 | 2.13 | 63 | 9.71 | 2.26 | 67 | 9.87 | 2.11 |
| TIPI - Conscientiousness | 258 | 10.19 | 2.88 | 128 | 10.07 | 3.07 | 63 | 10.49 | 2.61 | 67 | 10.15 | 2.75 |
| TIPI - Openness to Experience | 257 | 10.75 | 2.37 | 127 | 11.28 | 2.28 | 63 | 10.27 | 2.08 | 67 | 10.21 | 2.60 |
| TIPI - Emotional Stability | 258 | 8.54 | 3.06 | 128 | 8.23 | 3.15 | 63 | 8.79 | 2.89 | 67 | 8.79 | 3.04 |
